# Adipose tissue ATGL modifies the cardiac lipidome in pressure-overload-induced left ventricular failure

**DOI:** 10.1371/journal.pgen.1007171

**Published:** 2018-01-10

**Authors:** Janek Salatzki, Anna Foryst-Ludwig, Kajetan Bentele, Annelie Blumrich, Elia Smeir, Zsofia Ban, Sarah Brix, Jana Grune, Niklas Beyhoff, Robert Klopfleisch, Sebastian Dunst, Michal A. Surma, Christian Klose, Michael Rothe, Frank R. Heinzel, Alexander Krannich, Erin E. Kershaw, Dieter Beule, P. Christian Schulze, Nikolaus Marx, Ulrich Kintscher

**Affiliations:** 1 Charité-Universitaetsmedizin, Institute of Pharmacology, Center for Cardiovascular Research, Berlin, Germany; 2 DZHK (German Centre for Cardiovascular Research), partner site Berlin, Germany; 3 Berlin Institute of Health, BIH, Core Unit Bioinformatics, Berlin, Germany; 4 Max Delbrück Center for Molecular Medicine, MDC, Berlin, Germany; 5 Department of Veterinary Pathology, Freie Universität Berlin, Berlin, Germany; 6 Department of Experimental Toxicology and ZEBET, German Centre for the Protection of Laboratory Animals (Bf3R), German Federal Institute for Risk Assessment, Berlin, Germany; 7 Lipotype GmbH, Dresden, Germany; 8 Lipidomix GmbH, Berlin, Germany; 9 Department of Cardiology, Charité-Universitaetsmedizin, CVK, Berlin, Germany; 10 Coordination Center for Clinical Trials, Charité-Universitaetsmedizin/ BIH, Berlin, Germany; 11 Division of Endocrinology and Metabolism, University of Pittsburgh, Pittsburgh, Pennsylvania, United States of America; 12 Department of Internal Medicine I, Division of Cardiology, Angiology, Pneumology and Intensive Medical Care, University Hospital Jena, Friedrich-Schiller-University Jena, Germany; 13 Department of Internal Medicine I, Cardiology, Angiology, Pneumology and Internal Intensive Care Medicine, University Hospital, RWTH Aachen, Aachen, Germany; Stanford University School of Medicine, UNITED STATES

## Abstract

Adipose tissue lipolysis occurs during the development of heart failure as a consequence of chronic adrenergic stimulation. However, the impact of enhanced adipose triacylglycerol hydrolysis mediated by adipose triglyceride lipase (ATGL) on cardiac function is unclear. To investigate the role of adipose tissue lipolysis during heart failure, we generated mice with tissue-specific deletion of ATGL (atATGL-KO). atATGL-KO mice were subjected to transverse aortic constriction (TAC) to induce pressure-mediated cardiac failure. The cardiac mouse lipidome and the human plasma lipidome from healthy controls (n = 10) and patients with systolic heart failure (HFrEF, n = 13) were analyzed by MS-based shotgun lipidomics. TAC-induced increases in left ventricular mass (LVM) and diastolic LV inner diameter were significantly attenuated in atATGL-KO mice compared to wild type (wt) -mice. More importantly, atATGL-KO mice were protected against TAC-induced systolic LV failure. Perturbation of lipolysis in the adipose tissue of atATGL-KO mice resulted in the prevention of the major cardiac lipidome changes observed after TAC in wt-mice. Profound changes occurred in the lipid class of phosphatidylethanolamines (PE) in which multiple PE-species were markedly induced in failing wt-hearts, which was attenuated in atATGL-KO hearts. Moreover, selected heart failure-induced PE species in mouse hearts were also induced in plasma samples from patients with chronic heart failure. TAC-induced cardiac PE induction resulted in decreased PC/ PE-species ratios associated with increased apoptotic marker expression in failing wt-hearts, a process absent in atATGL-KO hearts. Perturbation of adipose tissue lipolysis by ATGL-deficiency ameliorated pressure-induced heart failure and the potentially deleterious cardiac lipidome changes that accompany this pathological process, namely the induction of specific PE species. Non-cardiac ATGL-mediated modulation of the cardiac lipidome may play an important role in the pathogenesis of chronic heart failure.

## Author summary

Chronic heart failure (CHF) is still one of the leading causes of morbidity and mortality worldwide, and new therapeutic approaches are required. Here we show that perturbation of adipose tissue lipolysis by tissue-specific deletion of the lypolytic enzyme ATGL (adipose trigylceride lipase) protects against heart failure development. By using MS-based lipidomics we show that the lack of ATGL in adipose tissue protects the heart against deleterious cardiac lipidome changes such as increases of phosphatidylethanolamines. Changes of cardiac phosphatidylethanolamines may contribute to adipose ATGL-mediated heart failure pathophysiology by an impairment of cell membrane integrity. These data point towards a crucial function of ATGL in adipose tissue by regulating processes outside of the primary target tissue such as the heart. This is a new concept which may lead to alternative studies of gene function with a focus on non-target tissue and tissue/ organ interactions. Furthermore, therapeutic targeting of non-cardiac tissue may provide new opportunities for future heart failure therapy.

## Introduction

The development of chronic systolic heart failure is marked by a continuous incline in adrenergic activity that results in the typical clinical signs of this disease, including tachycardia and increased peripheral vascular resistance [1, 2]. In addition to these cardiovascular effects, chronic sympathetic activation exerts crucial effects on adipose tissue metabolism, including a marked induction of adipose tissue lipolysis via β-receptor stimulation [3]. Adipose tissue lipolysis is further enhanced by natriuretic peptides such as atrial natriuretic peptide (ANP) and brain natriuretic peptide (BNP), both of which are elevated in chronic heart failure [4]. Together, these processes lead to a higher lipolytic rate in patients with heart failure compared to control subjects [5]. Increased adipose tissue lipolysis is associated with elevated plasma levels of free fatty acids (FFA) in patients with chronic heart failure [5, 6]. This ultimately leads to systemic metabolic disturbances such as insulin resistance and glucose intolerance, which further perpetuate the cycle of impaired left ventricular dysfunction [7, 8]. However, the immediate consequences of stimulated adipose tissue lipolysis on cardiac morphology and function during heart failure development are largely unknown.

Triacylglycerol (TAG) hydrolysis in adipose tissue occurs in a stepwise process and involves three major lipases: adipose triglyceride lipase (ATGL), hormone sensitive lipase (HSL), and monoglyceride lipase (MGL), the cumulative effect of which is an efflux of glycerol and FFA from adipocytes into the circulation [3]. ATGL, also referred to as patatin-like phospholipase containing A2 (PNPLA2), catalyzes the first step of TAG hydrolysis to DAGs [3]. HSL has broad substrate specificity and mediates the hydrolysis of TAGs to diacylglycerols (DAGs), and DAGs to monoacylglycerols (MAGs), as well as the cleavage of FAs from MAGs [3]. Of these three functions, HSL is most efficient at regulating DAG hydrolysis [3]. Finally, MGL mediates the hydrolysis of MAGs to glycerol and FAs [3].

Mice carrying a constitutive deletion of ATGL showed a marked reduction of FA release from white adipocytes and of TAG accumulation in adipose tissue [9]. Similar findings were observed in mice with conditional ATGL-deletion in adipose tissue demonstrating reduced adipocyte lipolysis, decreased FA release from white adipose tissue associated with decreased non-esterified FA- and TAG-blood levels, and impaired brown adipose tissue function [10, 11]. Moreover, the deletion of ATGL in adipocytes markedly improved hepatic insulin signaling and attenuated inflammatory reactions in the liver, confirming the presence of crosstalk between adipose tissue and the liver [11]. These data corroborate the important role of adipose tissue ATGL in the regulation of blood lipid levels, and the potential of this enzyme to affect the function of non-adipose tissue organs, particularly under conditions of increased lipolysis (e.g. fasting, exercise, heart failure). Along this line, we recently demonstrated that deletion of ATGL in adipose tissue attenuated the development of exercise-induced physiological cardiac hypertrophy in female mice [12]. More importantly, perturbation of adipose tissue lipolysis by ATGL deficiency modified the circulating lipid profile, and a selected FA, namely C16:1 palmitoleic acid, was identified as a new molecular mediator of training-induced cardiac responses [12]. Interorgan communication between adipose tissue and other organs has been recently described as a crucial regulator of insulin action. In accordance with our results, Cao and colleagues identified C16:1 palmitoleic acid as a lipokine released from adipose tissue modulating insulin sensitivity in muscle and liver [13]. Thus, communication between adipose tissue and other organs appears to be a common process during the initiation and progression of different diseases [14].

In this study, we report that perturbation of adipose tissue lipolysis by conditional deletion of ATGL protects against pressure-induced cardiac failure. By applying an untargeted shotgun lipidomics approach, we identified cardiac lipidome changes significantly associated with LV failure and demonstrated that these were prevented by ATGL deficiency in adipose tissue. In particular, failing wild-type (wt) hearts exhibited a marked increase of multiple phosphatidylethanolamines (PE) (PE16:0–18:1, PE16:0–18:2, PE16:0–20:4, PE17:0–20:4, PE18:0–20:4, PE18:0–22:4, PE18:1–18:1, PE18:2–18:0, PE18:2–19:0, PE20:4–20:0) whereas ceramides (Cer) (Cer36:1; Cer38:1; Cer38:2), and cardiolipin (CL76:12) were decreased. These changes were prominently attenuated in atATGL-KO hearts. Moreover, distinct heart-failure-regulated PE species in mouse hearts were modified in a similar manner in plasma samples from patients with chronic heart failure. These findings strongly suggest that adipose tissue lipolysis determines cardiac morphology and function during heart failure development. This inter-organ crosstalk likely involves an alteration of the cardiac lipidome which may mediate functional impairment by mechanisms such as induction of apoptosis or mitochondrial dysfunction.

## Results

### Deletion of ATGL in adipose tissue prevents TAC-induced heart failure

We previously described that fabp4-Cre-driven ATGL deletion resulted in a lack of ATGL in white adipose tissue but not in the heart or in bone marrow-derived macrophages, and in an attenuation of adipose tissue lipolysis [12]. To exclude that the fabp4-Cre mediates deletion of ATGL in endothelial cells, as previously described [15], we analyzed ATGL expression in cardiac endothelial cells. ATGL expression did not significantly differ between wt- and atATGL-KO mice (S1A Fig).

**Fig 1:**
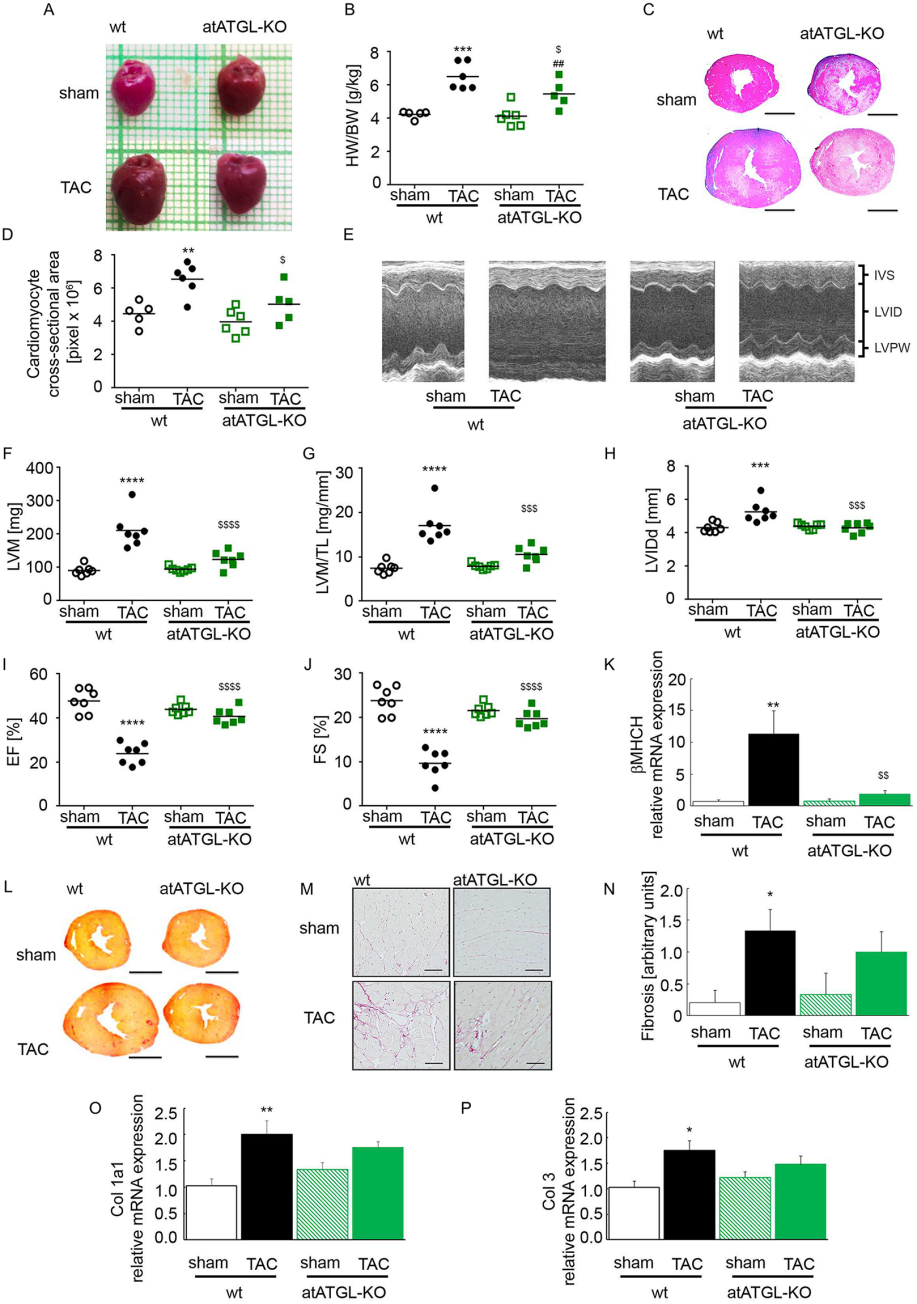
Deletion of ATGL in adipose tissue attenuates pressure overload-induced LV failure. **A:** Representative images of the hearts. **B**: Heart weight (HW)/ body weight (BW) ratio (mean and SEM, n = 5–6). **C**: Representative microscopic cross-sections of the hearts stained with hematoxylin/ eosin (H/E). **D**: Myocardial area, calculated based on microscopic sections of heart tissue, stained with H/E, analogue to the images presented in C (mean and SEM, n = 5–6). **E**: Representative M-Mode images of the echocardiographic analysis. **F-J**: cardiac echocardiographic analysis of mice (mean and SEM, n = 7): **F**: Left-ventricular mass (LVM). **G**: LVM relative to tibia length (LVM/TL). **H**: Left-ventricular internal diameter in diastole (LVID-d). **I**: Ejection fraction [%] (EF). **J**: Fractional shortening [%] (FS). **K**: Analysis of mRNA expression of beta-cardiac myosin heavy chain isogene (βMHCH), qRT-PCR studies were carried out using total RNA isolated from LV tissue. Data are presented as x-fold over wt-sham mice (mean and SEM, n = 5–6). **L**: Representative microscopic cross-sections of the hearts stained with picrosirius red. **M**: Representative high magnification images from picrosirius red-stained sections. **N:** Cardiac fibrosis calculated based on microscopic sections of the heart tissue, stained with picrosirius red, analogue to the images presented in L: 0 = no fibrosis, 1 = mild fibrosis, 2 = moderate fibrosis, 3 = severe fibrosis (mean and SEM, n = 5–6). **O and P:** Analysis of mRNA expression of collagen (Col) 1a1 (O) and Col3 (P), qRT-PCR studies were carried out using total RNA isolated from LV tissue. Data are presented as x-fold over wt-sham mice (mean and SEM, n = 5–6).*p<0.05 vs. wt sham, **p<0.01 vs. wt sham, ***p<0.001 vs. wt sham, ****p<0.0001 vs. wt sham, $ p<0.05 vs. wt TAC, $ $ p<0.01 vs. wt TAC, $ $ $ p<0.001 vs. wt TAC, $ $ $ $ p<0.0001 vs. wt TAC, ##p<0.01 vs. atATGL-KO sham; 2-way ANOVA (Bonferroni post-test).

Mice were challenged with transverse aortic constriction (TAC) to induce pressure-mediated cardiac enlargement or failure. Pressure gradients (pre-/ post-stenotic) in TAC mice assessed by Doppler echocardiography did not differ between wt- and atATGL-KO mice, indicating similar degree of stenosis, LV-pressure load and aortic vascular function in both genotypes (wt-TAC: 54.2±2.5mmHg vs. atATGL-KO-TAC: 48.1±3.4mmHg; p = n.s.). Eleven weeks post TAC, wt hearts increased markedly in size and weight compared to wt-sham mice (Fig 1A and 1B). TAC-induced cardiac enlargement was attenuated in atATGL-KO mice (Fig 1A and 1B). Accordingly, the myocardial area in cardiac cross section increased significantly in wt-mice after TAC whereas this effect was diminished in atATGL-KO mice (Fig 1C and 1D). Echocardiographic analysis revealed a significant rise in left ventricular mass (LVM) in wt-mice 11 weeks after TAC (Fig 1E–1G and S1 Table). The induction of LVM by pressure overload was clearly suppressed in atATGL-KO mice (Fig 1E–1G and S1 Table). In addition, pressure-induced LV enlargement in wt-mice shown by the increased diastolic LV inner diameter (LVIDd) was completely prevented in atATGL-KO mice (Fig 1H and S1 Table). To assess LV function after TAC, LV ejection fraction (EF) and fractional shortening (FS) were analyzed in wt and atATGL-KO mice (Fig 1I and 1J). In wt-mice, pressure-overload led to a significant reduction in EF and FS after 11 weeks (Fig 1I and 1J), but this was not observed in atATGL-KO mice (Fig 1I and 1J). In keeping with the morphological changes described above, marker genes for cardiac failure such as the beta-myosin heavy chain (β-Myhc) were also markedly induced by TAC in wt-hearts but not in atATGL-KO hearts (Fig 1K). Cardiac fibrosis was significantly induced during pressure overload in wt-mice as shown by collagen fiber staining (Fig 1L–1N) and mRNA expression of the fibrotic marker genes, collagen (Col) 1a1 and 3 (Fig 1O and 1P). In atATGL-KO mice we observed a moderate induction of collagen fibers (Fig 1L–1N) and fibrotic marker genes (Fig 1O and 1P) by TAC, however, this did not reach statistical significance (Fig 1N) suggesting attenuated pressure-induced fibrotic remodeling in the absence of atATGL. Together, these data show that perturbation of ATGL in adipose tissue protects the heart against pressure-induced LV enlargement and systolic failure.

### Deletion of adipose ATGL alters circulating FA levels and the cardiac lipidome during pressure overload

**Fig 2:**
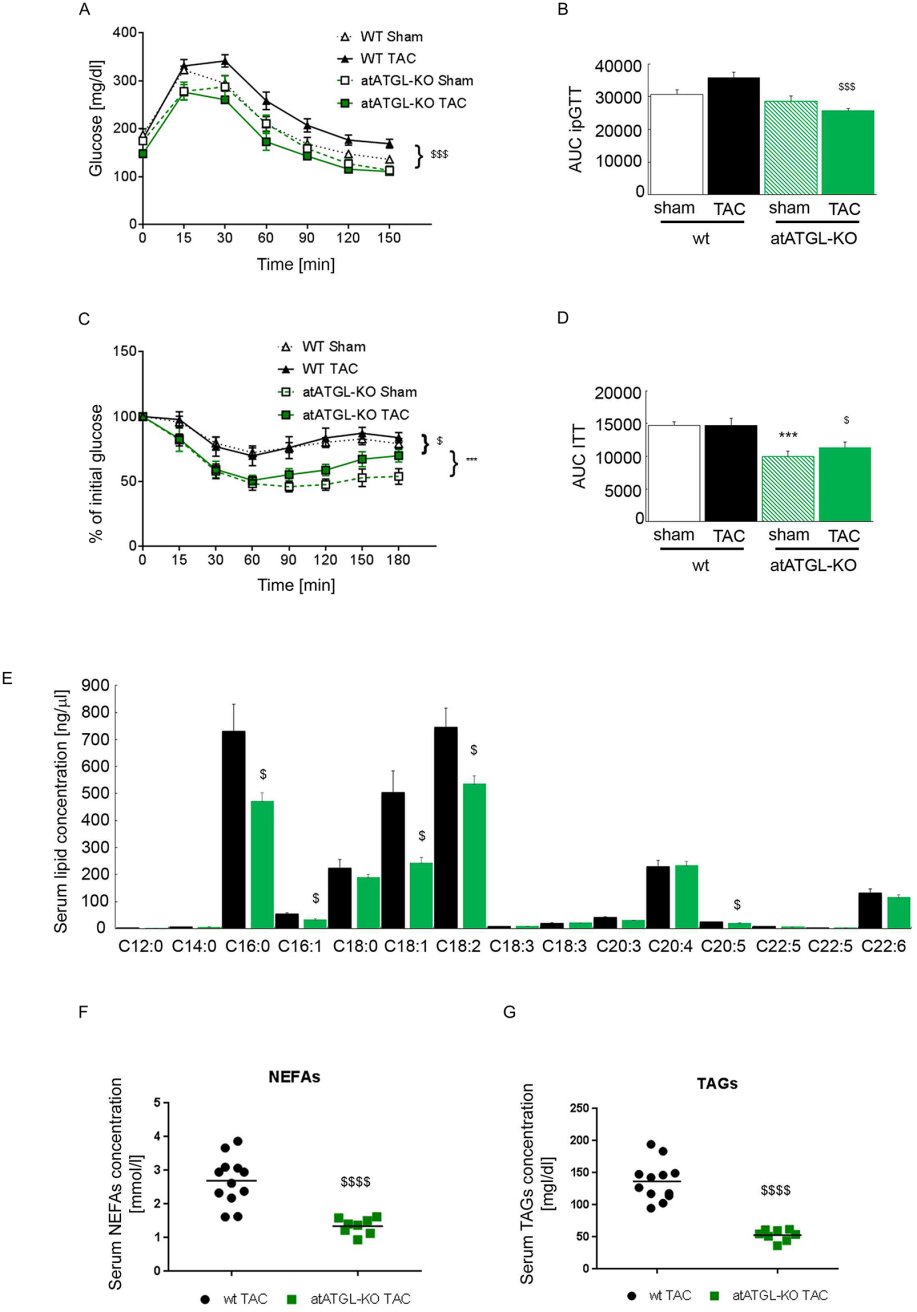
Metabolic phenotype and analysis of blood FA-profile in wt- and atATGL-KO mice. **A:** Intraperitoneal Glucose Tolerance Test (ipGTT), (n = 4–6, 2-way ANOVA (Bonferroni post-test) from AUC). **B**: Area under the curve (AUC) of ipGTT, (mean and SEM, n = 4–6, 2-way ANOVA (Bonferroni posttest)). **C**: Insulin Tolerance Test (ITT), (n = 7–8, 2-way ANOVA (Bonferroni posttest) from AUC). **D**: Area under the curve (AUC) of ITT, (mean and SEM, n = 7–8, 2-way ANOVA (Bonferroni posttest)). **E**: Profile of selected serum FAs in TAC-operated mice analyzed by rapid resolution HPLC/ Tandem MS. **F**: Serum level of non-esterified fatty acids (NEFAs) in wt-TAC and atATGL-KO-TAC mice. **G**: Serum level of triacylglyerols (TAGs) in wt-TAC and atATGL-KO-TAC mice. (mean and SEM, n = 5, or as indicated, unpaired t-test). ***p<0.001 vs. wt sham, $ p<0.05 vs. wt TAC, $ $ $ p<0.001 vs. wt TAC, $ $ $ $ p<0.001 vs. wt TAC.

Reduced adipose tissue lipolysis in atATGL-KO mice led to improved insulin sensitivity and glucose tolerance compared to their wt littermates (Fig 2A–2D). In addition, perturbation of adipose ATGL abrogated TAG-hydrolysis leading to a diminished release of glycerol and FAs in the circulation [3]. Accordingly, multiple TAG species were significantly increased in white adipose tissue from atATGL-KO mice compared to wt littermates (S1B Fig). Primarily, TAGs with shorter FA chain length and lower desaturation grade were significantly regulated in atATGL-KO mice. This is in accordance with ATGL´s substrate selectivity which declines with increasing FA chain length and desaturation [16]. To identify potential factors involved in the regulation of cardiac function in atATGL-KO mice, we next examined whether the lack of ATGL regulates the release of preferential FAs from adipose tissue in a selective manner. Our analysis measured the total amount of FAs present in serum and not the amount of free circulating FAs. After alkaline hydrolysis, the total serum concentration of distinct FAs (independent of their head group) was analyzed using HPLC coupled with a triple quad mass spectrometer with electrospray ionization. As depicted in Fig 2E (TAC) and S1C Fig (sham), lack of ATGL in adipose tissue resulted in a significant reduction of selected circulating FAs including C16:0 (palmitic acid), C16:1 (palmitoleic acid), C18:1 (oleic acid), C18:2 (linoleic acid), and C20:5 (eicospentanoic acid) in TAC mice (Fig 2E). In accordance, non-esterified FA (NEFA) and TAG serum levels were also significantly lower in atATGL-KO mice compared to wt-mice (Fig 2F and 2G).

We hypothesized that dysregulated serum FAs may serve as the biochemical bridge between the lack of ATGL in adipose tissue and the observed cardiac phenotype. Thus, we next investigated whether modifications of the circulating FA profile are accompanied by changes in the cardiac lipidome using MS-based shotgun lipidomics.

**Fig 3:**
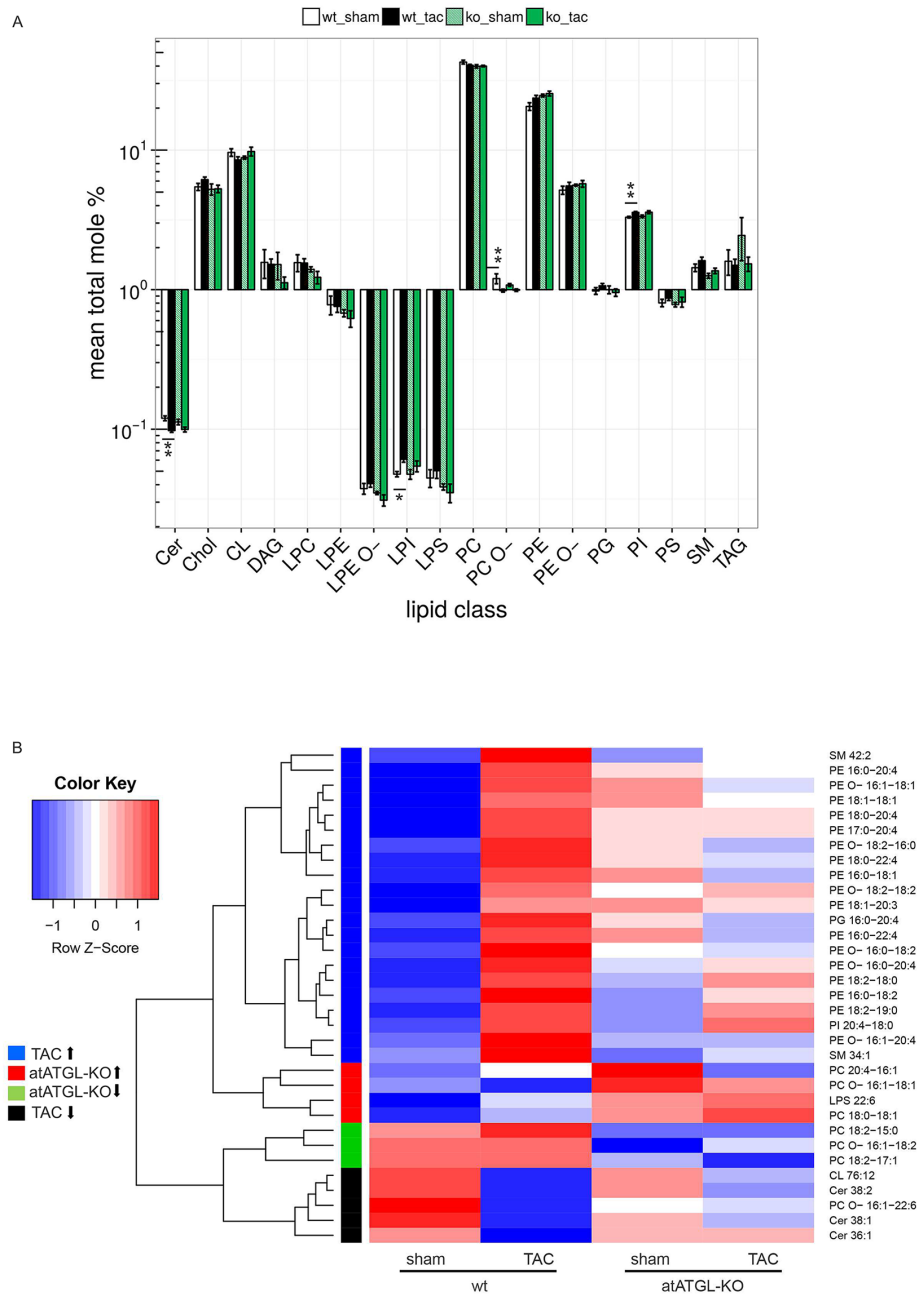
Perturbation of adipose ATGL alters the cardiac lipidome during pressure overload. MS-based shotgun lipidomics analysis of heart tissue samples (LV) isolated 11 weeks after intervention (sham or TAC) from wild-type (wt) or atATGL-KO mice. **A:** For each lipid class, mean total mole percent values ± SEM are shown on a logarithmic scale. To test for differential changes, a Mann-Whitney U test between sham and TAC in wt- and atATGL-KO mice, respectively, was performed. Adjusted p-values are indicated: *p<0.05 vs. wt sham, **p<0.01 vs. wt sham. **B:** Differentially regulated lipid species were filtered by testing for the effect of the genotype (wt; atATGL-KO), the intervention (sham; TAC) and the sum of both plus their interaction using a linear model. Species with an FDR adjusted p-value < 0.1 in at least one test were retained. Hierarchical clustering of the corresponding t-statistics yielded four distinct groups: lipid species upregulated in TAC (blue), upregulated in atATGL-KO (red), downregulated in atATGL-KO (green), downregulated in TAC (black). Data for each lipid species are presented as row scaled z-scores of mean log-transformed relative changes. Lipid classes: Cer: ceramide, Chol: cholesterol, CL: cardiolipin, DAG: diacylglycerol, LPC: lyso-phosphatidylcholine, LPE: lyso-phosphatidylethanolamine, LPE O-: lyso-phosphatidylethanolamine-ether, LPI: lyso-phosphatidylinositol, LPS: lyso-phosphatidylserine, PC: phosphatidylcholine, PC O-: phosphatidylcholine-ether, PE: phosphatidylethanolamine, PE O-: phosphatidylethanolamine-ether, PG: phosphatidylglycerol, PI: phosphatidylinositol, PS: phosphatidylserine, SM: sphingomyelin, TAG: triacylglycerol.

A total of 225 lipid species from 18 different lipid classes were analyzed in LV samples, and included glycerolipids, glycerophospholipids, sterol lipids and sphingolipids (S2 Table). The most abundant lipid classes in mouse hearts were PCs, PEs, and CLs (Fig 3A). Comparison of lipid classes in each genotype revealed significant changes in levels of the low-abundant classes of Cers, LPIs, PC O-s, and PIs in failing wt-hearts vs. sham controls (Fig 3A). These changes were not significant in atATGL-KO mice (Fig 3A). All other classes were not significantly altered by the intervention (Fig 3A). To compare the abundance of individual lipid species between the 4 groups, we used a linear model and performed three tests to capture the effects of the genotype (wt; atATGL-KO), the intervention (sham; TAC) and the sum of both and their interaction. Multiple testing correction was applied, and all lipid species with a significant p-value (adjusted) in at least one test are shown in the heat map (Fig 3B). Data for each lipid species are presented as row z-scores of the mean log2-transformed relative values. The complete data set of the analysis is shown in S2 Table.

Among the identified lipid species, the most significant change was an increase in individual PE species in failing wt-hearts (TAC) compared to wt-sham hearts (Fig 3B). This increase was clearly attenuated in mice lacking adipose ATGL comparing atATGL-KO TAC mice and their sham controls (Fig 3B). A similar effect was detected for two sphingomyelins (SM), SM34:1 and SM42:2, for phosphatidylglycerol (PG), PG16:0–20:4, and for phosphatidylinositol (PI), PI20:4–18:0 (Fig 3B). In contrast, other lipid species were markedly downregulated in failing hearts from wt-mice, an effect broadly extenuated in atATGL-KO mice (Fig 3B). The specific lipid species reduced by pressure-overload in wt-mice included PC O 16:1–22:6, Cers (Cer36:1; Cer38:1; Cer38:2), and the CL76:12 (Fig 3B). This downregulation was attenuated in atATGL-KO mice (Fig 3B). Comparison of wt- and atATGL-KO mice independent of the intervention revealed primarily changes of a small number of lipid species including the species from the PC class: upregulation of PC20:4–16:1 and PC18:0–18:1 and downregulation of PC18:2–15:0 and PC18:2–17:1 (Fig 3B).

In summary, lack of ATGL in adipose tissue and reduced lipolysis associated with the reduction of selected circulating FAs mediates pressure-induced modifications of distinct cardiac lipid species among which PEs display the most apparent regulation.

### Induction of pressure-mediated cardiac PE species is attenuated in atATGL-KO mice

**Fig 4:**
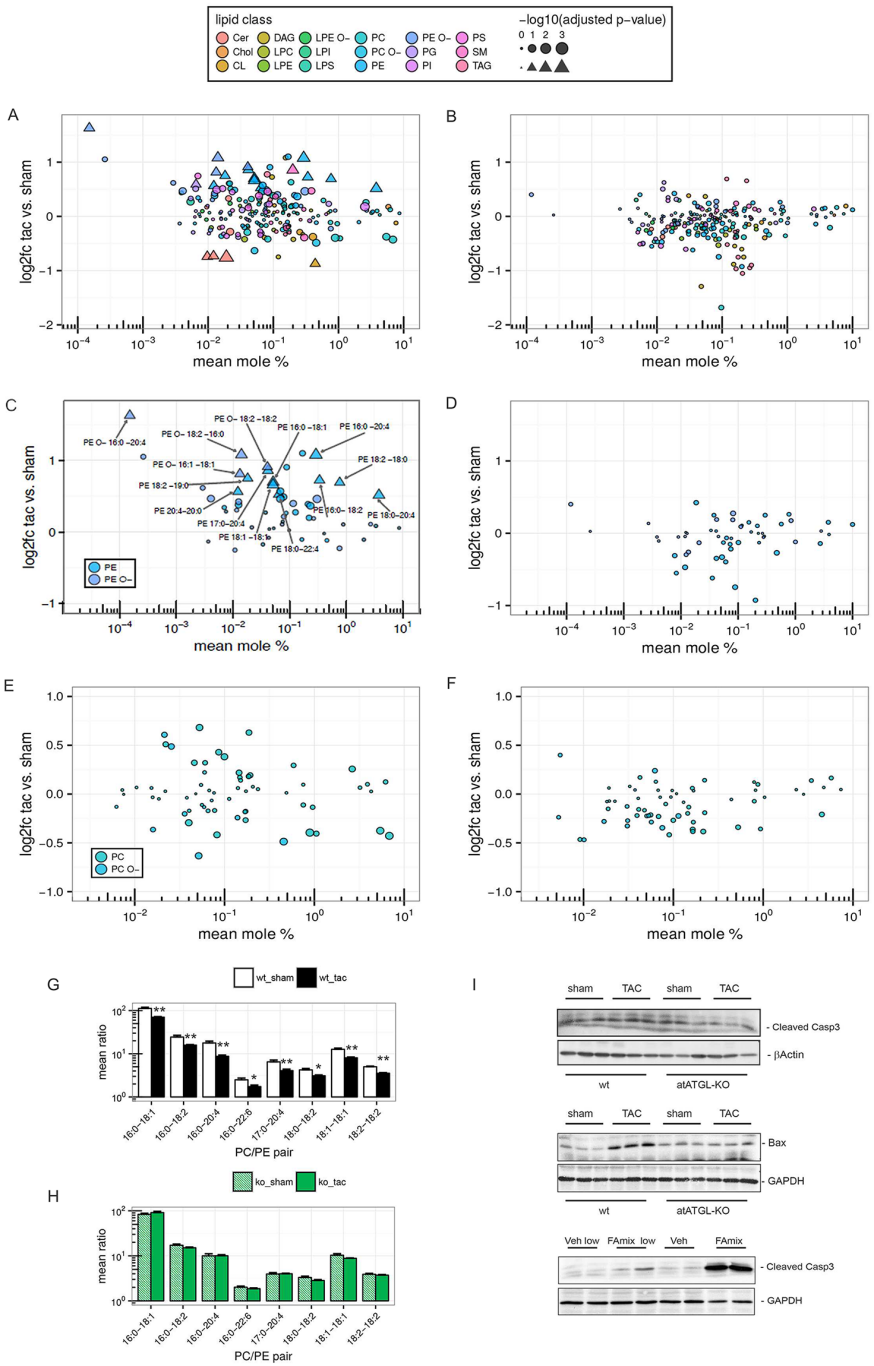
Induction of pressure-mediated cardiac PE species is attenuated in atATGL-KO mice. MS-based shotgun lipidomics analysis of heart tissue samples (LV) isolated 11 weeks after intervention (sham or TAC) from wild-type (wt) or atATGL-KO mice. Lipid class denotations see Fig 3. **A-F:** Mean log2-fold change (TAC vs. sham) vs. mean mole percent of lipid species. Triangles represent significantly changed lipid species (FDR adjusted p-value < 0.1 and absolute value of log2-fold change ≥ 0.5); bubbles represent those which are not significantly changed; size indicates log-transformed adjusted p-values. **A, C, E:** wt-mice. **B, D, F:** atATGL-KO-mice. **G+H:** Significantly changed PC-PE ratios of matched FAs in wt-mice (**G**) or atATGL-KO mice (**H**). The mean ratio ± SEM is shown on a logarithmic scale, Mann-Whitney U test for TAC vs. sham: *p<0.05, **p<0.01 (adjusted) in wt-mice, no significant changes were found in at ATGL-KO mice. **I:** upper panels: WB analysis of heart lysates using antibodies against cleaved caspase 3 and Bcl-associated X protein (Bax); lower panel: WB analysis of HL-1 cardiomyocytes lysates from cells stimulated with vehicle (Veh) or fatty acid (FA) mix (C16:0, C18:1,C18:2 in different concentrations) using antibodies against cleaved caspase 3; loading control: β–actin, glyceraldehyde 3-phosphate dehydrogenase (GAPDH).

In order to better understand the degree and relevance of cardiac lipid changes during LV failure, we performed an in-depth analysis of all lipid classes in which the log2-fold change (sham vs. TAC), and the concentration of each lipid species (mean mole %) was included in a pairwise comparison (sham vs. TAC) in wt and atATGL-KO mice. Fig 4A and 4B provides an overview of all lipid classes and species in wt and atATGL-KO mice, respectively. The overall extent of regulation of cardiac lipid species in sham vs. TAC mice was obviously greater in wt-mice compared to atATGL-KO mice, as reflected by the vertical distribution pattern on the scatter plot (Fig 4A and 4B). TAC-induced regulation of lipid species in wt-hearts (Fig 4A, triangles) was robustly attenuated in atATGL-KO mice (Fig 4B). Detailed evaluation of each regulated lipid class revealed a total of 10 PE-species significantly upregulated during LV failure in wt-hearts containing mostly saturated, mono- or polyunsaturated long-chain FAs (Fig 4C). The PE with the highest concentration in wt LV samples (PE18:0–20:4) exhibited an induction of 1.4-fold over sham controls (Fig 4C). The maximum PE induction by TAC intervention was 2.1-fold over sham for PE16:0–20:4. Some PE-species in the higher concentration range were not significantly regulated by TAC (Fig 4C, circles), likely explaining the absence of a statistical significant regulation of the total PE class, as shown in Fig 3A. Induction of significantly regulated PEs during heart failure development was absent in atATGL-KO mice (Fig 4D). In addition, we identified 4 ether-PEs (PE O-) containing a fatty alcohol instead of a fatty acid at the sn1-position which were upregulated in failing wt-hearts but not in atATGL-KO hearts (Fig 4C and 4D). Among the PE O-s, PE O- 16:0–20:4 showed the most dramatic induction over sham controls in wt mice (3.1-fold over sham) (Fig 4C). In accordance to the diacyl PE regulation, hearts from atATGL-KO mice lacked the significant PE O- induction after TAC (Fig 4D). The majority of the differentially regulated PE- and PE O- species in failing wt-hearts contained at least one FA from the depleted blood FA pool (C16:0, C16:1; C18:1, C18:2, C20:5, Fig 2E) in atATGL-KO mice (Fig 4C), suggesting a link between blood FAs and cardiac lipid species in the present model. In contrast to the PE- and PE O species, PC- and PC O- species were not significantly regulated in failing wt-hearts, or in atATGL-KO hearts (Fig 4E and 4F).

Recently, a decrease of the PC/ PE ratio has been identified to regulate cell membrane integrity in hepatocytes, and was accompanied by pronounced cell damage [17]. Thus, we calculated the ratio of the identified PCs and PEs matched for bound FAs in our model. In agreement with the decline of LV function in TAC-operated wt-mice, cardiac PC/ PE-ratios were reduced in failing wt-hearts compared to wt-sham hearts (Fig 4G). In atATGL-KO mice, PC/ PE ratios did not significantly differ between sham- and TAC-mice (Fig 4H). The highest ratio was detected in wt-sham hearts (S3 Table). Since the decrease of PC/ PE ratios has been associated with cell damage, we evaluated the expression of the pro-apoptotic markers cleaved caspase 3 and Bax in LV samples from sham- and TAC-operated wt- and atATGL-KO mice. As shown in Fig 4I (upper panels), both proteins were induced in failing LVs from wt-mice but not atATGL-KO mice. In accordance, when HL-1 cardiomyocytes were stimulated with FAs (C16:0, C18:1, C18:2) regulated by ATGL in-vivo, we detected increased apoptosis by cleaved caspase 3 protein expression (Fig 4I lower panel). Cleaved caspase 3 was not induced in HL-1 cells under physiological high serum conditions (S1D Fig) suggesting that increased FA-levels are required for the induction of apoptosis in these cells.

In summary, these data demonstrate that pressure-mediated LV failure is associated with an increase of distinct cardiac PE-species leading to a reduction of cardiac PC/PE ratios. The decline of PC/ PE ratios is accompanied by the enhanced expression of cardiac apoptosis markers. Deficiency of adipose tissue ATGL prevents cardiac PE upregulation in an FA-specific manner and protects the heart against LV failure.

### PE species are regulated in plasma samples from patients with HFrEF

To explore the relevance of our findings to LV failure in humans, we performed shotgun lipidomics analysis using plasma samples obtained from male patients with HFrEF (n = 13) and male control subjects with normal LV function (n = 10). The clinical characteristics of both groups are outlined in Table 1 and S4 Table. Patients with HFrEF had a mean LVEF of 25 ± 4.8% compared to 60.9 ± 3.3% in the control group (Table 1). HFrEF patients were on average older than subjects in the control group and had a slightly higher BMI (Table 1).

**Table 1:** 

Parameters	control subjects n = 10	HFrEF patients N = 13
Age [years]	43.3 [25–81]	59.2 [24–81]
Height [cm]	179.4 ± 6.6	177.6 ± 8.1
Weight [kg]	81.8 ± 6.5	86.0 ± 12.2
Body mass index [kg/m^2^]	25.5 ± 2.7	27.3 ± 3.6
previous MI [%]	0/10 [0]	4/13 [30.8]
Previous stroke [%]	0/10 [0]	2/13 [15.4]
LV ejection fraction [%]	60.9 ± 3.3	25.0 ± 4.8

Shown are means ± SD, [min.-max.], or [%]; MI: myocardial infarction; LV: left ventricular

**Fig 5:**
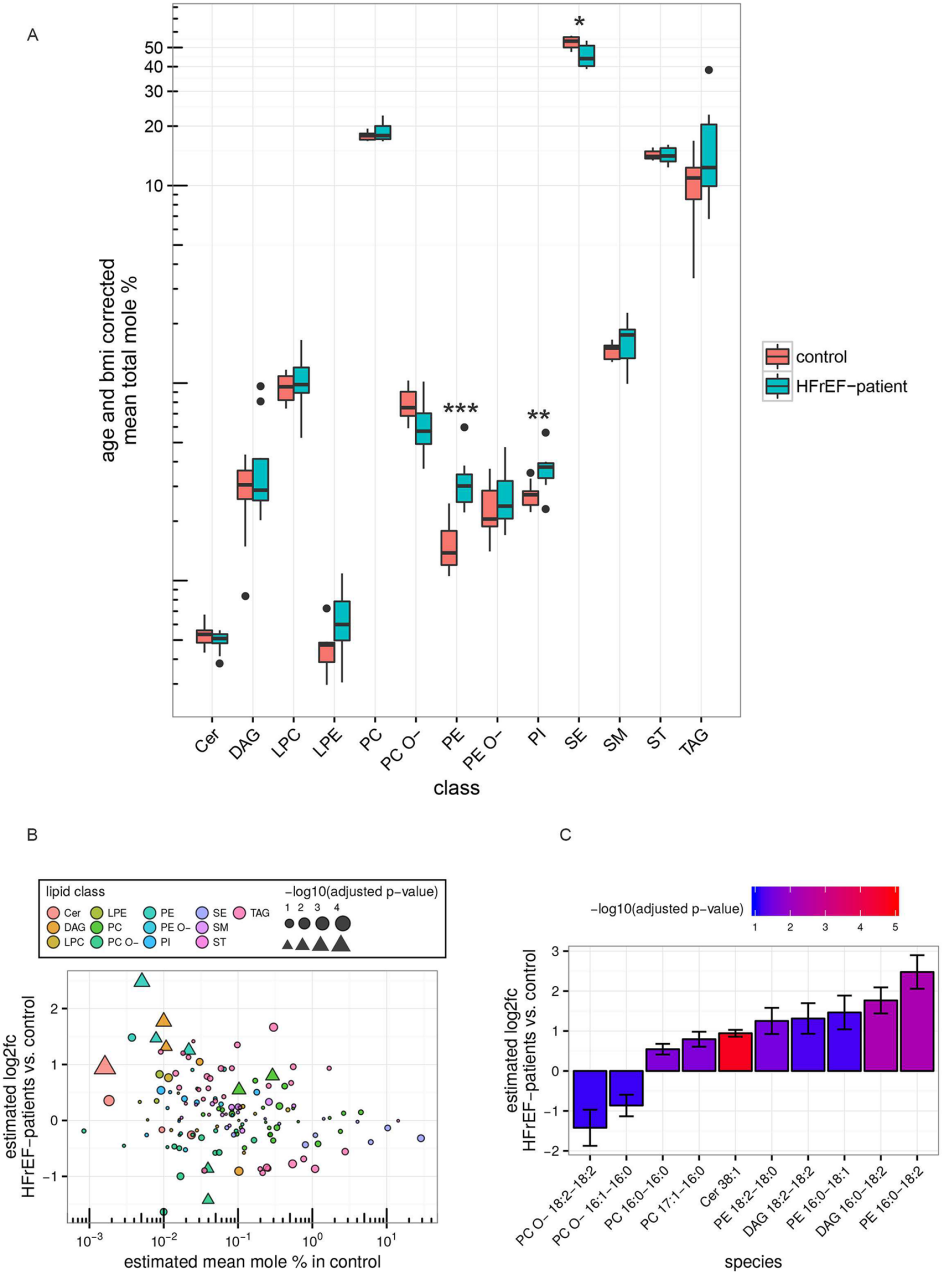
Selected lipid species are altered in plasma samples from patients with HFrEF MS-based shotgun lipidomics analysis of human plasma samples from HFrEF-patients (n = 13) and non-HFrEF controls (n = 10). A: Box plots show distribution of total mole percent values for each lipid class corrected for age and BMI (see supplementary methods). To test for differential changes a Mann-Whitney U test between HFrEF-patients and controls was performed. Adjusted p-values are indicated: *p<0.05, **p<0.01, **p<0.001. B: Estimated mean log2 fold change (HFrEF vs. control) vs. estimated mean mole percent of lipid species in the control group (see supplementary methods). Triangles represent significantly changed lipid species (FDR adjusted p-value < 0.1 and absolute value of log2-fold change ≥ 0.5), bubbles show those which are not significantly changed, size indicates log-transformed adjusted p-values. C: Bar graph shows the estimated log2-fold change (HFrEF vs. control) ± regression standard error of differentially changed lipid species (see B.). Colors represent log10 adjusted p-values as indicated. Lipid classes: Cer: ceramide, DAG: diacylglycerol, LPC: lyso-phosphatidylcholine, LPE: lyso-phosphatidylethanolamine, PC: phosphatidylcholine, PC O-: phosphatidylcholine-ether, PE: phosphatidylethanolamine, PE O-: phosphatidylethanolamine-ether, PI: phosphatidylinositol, SE: sterol ester, SM: sphingomyelin, ST: sterols, TAG: triacylglycerol.

We analyzed 147 lipid species from 13 lipid classes in human plasma (S5 Table and Fig 5A). PCs, TAGs, sterols (ST) and sterol esters (SE) were found in the highest concentration in human plasma (Fig 5A). HFrEF patients had significantly higher plasma levels of PEs and PIs, and lower plasma levels of SE compared to controls (Fig 5A). We subsequently analyzed the individual lipid species within a lipid class and detected 10 plasma lipid species which were significantly differentially regulated between controls and HFrEF patients using a linear model to account for age and BMI (Fig 5B, triangles, and Fig 5C). Among the regulated lipid species, 3 PE species (PE16:0–18:1, PE16:0–18:2, PE18:2–18:0) were significantly higher in blood plasma from HFrEF patients compared to controls (Fig 5B and 5C, S5 Table). Interestingly, all of the PE species with increased levels in blood plasma from HFrEF patients were also upregulated in the hearts of wt mice with LV failure (Fig 4C), suggesting a link between circulating and cardiac PE levels. The strongest rise was observed for PE16:0–18:2, with 5.6-fold higher levels in HFrEF compared to controls (S5 Table and Fig 5B and 5C). In line with the increase in PEs, distinct DAG species (which are known precursors of PEs) were higher in HFrEF patients including DAG16:0–18:2 and DAG18:2–18:2 (S5 Table and Fig 5B and 5C). In contrast to the mouse data, we discovered two PC species (PC16:0–16:0, PC17:1–16:0) and Cer38:1 upregulated, and 2 PC O- species (PC O- 16:1–16:0, PC O- 18:2–18:2) downregulated in plasma from HFrEF patients (S5 Table and Fig 5B and 5C). PC O- species were depleted from the same FAs (C16:0; C16:1, and C18:2) which were concurrently increased in PE species, suggesting a possibility of selective FA partitioning to distinct glycerophospholipid classes in HFrEF (S5 Table and Fig 5B and 5C).

Taken together, our data from human plasma samples demonstrate that the presence of HFrEF is accompanied by a dysregulation of individual lipid classes and lipid species. Interestingly, comparable changes in PE species were observed in failing mouse hearts and in plasma from HFrEF patients.

## Discussion

In this study, we demonstrated for the first time that pressure-induced cardiac failure is accompanied by marked changes in the cardiac lipidome. The most robust alteration in lipid species was an upregulation of distinct PE species in failing wt-hearts. Perturbation of adipose tissue lipolysis in atATGL-KO mice led to an improvement of pressure-induced heart failure accompanied by the prevention of cardiac PE induction. Pressure-induced cardiac PE induction resulted in a decreased PC/ PE-ratio and a concurrent increase in apoptotic marker expression in failing wt-hearts, a process which was absent in atATGL-KO hearts. Taken together, this study demonstrates that the cardiac lipidome undergoes profound changes during heart failure development. The absence of these changes in atATGL-KO mice suggests that non-cardiac tissues such as adipose tissue, and in particular ATGL-mediated adipose tissue lipolysis, participate in the regulation of the cardiac lipidome.

The role of ATGL in heart failure development and cardiac metabolism has been previously studied, with most studies focusing mainly on cardiac ATGL function [18–20]. In this study, we concentrated on adipose ATGL and the impact of adipose tissue lipolysis on the development of pressure-induced LV failure. Whereas previous studies demonstrated that perturbation of cardiac ATGL resulted in the accumulation of TAG and impaired systolic LV function [9, 18, 21], we showed that the deletion of ATGL in adipose tissue prevents pressure-induced LV failure. This finding suggests that adipose tissue lipolysis has a deleterious effect during heart failure development. These data are in accordance with the positive association between augmented adipose tissue lipolysis and impairment of LV function in heart failure [5, 6]. The relevance of adipose tissue lipolysis in heart failure development has recently been supported by a study in mice deficient for the adipose-specific perilipin-1 isoform [22]. Perilipin-1 has been shown to prevent TAG hydrolysis in adipocytes [22, 23]. The lack of perilipin-1 in these mice resulted in enhanced fat tissue lipolysis associated with the development of hypertrophic cardiomyopathy and LV failure [22]. Together, these data support a role for adipose tissue lipolysis in the pathogenesis of LV failure.

Communication between the heart, the vasculature and other organs has been recently described as an important pathophysiological mechanism involved in the development of cardiovascular disease [24]. Cross-talk between skeletal muscle—heart, or between kidney—heart/ vasculature has gained increasing attention in the treatment of diseases such as cardiac cachexia or the cardiorenal syndrome [24, 25]. Adipose tissue communicates with multiple organs such as liver and skeletal muscle [14], or as described in this study with the heart. Whether this cross-talk will result in new therapies for heart failure requires additional studies. Recently, Schweiger and colleagues characterized a new small molecule inhibitor of ATGL, named Atglistatin, in a mouse model of diet-induced obesity [26]. They described that Atglistatin preferentially inhibits lipolysis in adipose tissue thereby improving weight gain, insulin resistance and liver steatosis, demonstrating that an adipose tissue-based pharmacological therapy of non-adipose organs might be feasible. [26].

Lipidomic analysis using advanced MS-technologies has been recently utilized in cardiovascular research for the identification of new biomarkers in the context of risk prediction [27]. Stegemann and colleagues [27] identified plasma TAGs and CEs with short acyl chains and low degree of unsaturation, such as TAG54:2 and CE16:1, as strong predictors for cardiovascular disease in the Bruneck cohort. In contrast to the analysis in blood plasma, data on lipid profiling in cardiac tissue are very limited and have focused primarily on lipid class analysis. These data have shown that in patients with heart failure and obesity or diabetes, cardiac TAG levels are increased [28]. In contrast, in the absence of metabolic disease, cardiac TAG levels were significantly decreased in patients with advanced heart failure undergoing left ventricular assist device (LVAD) implantation, while Cers and DAGs were increased [29]. In a recent review, Schulze and colleagues [30] concluded from this that cardiac lipotoxicity in heart failure is primarily driven by Cers and DAGs and not TAGs. In the present study, Cers, DAGs, and TAGs were not significantly increased in failing hearts from wt-mice or in human plasma. On the contrary, cardiac Cer levels were mildly reduced in failing LVs from wt-mice. One explanation for this discrepancy might be the absence of systemic insulin resistance in our study. Systemic insulin resistance was significantly induced in patients with heart failure in the LVAD study [29], whereas in our model, pressure overload-mediated LV failure was not accompanied by an impairment of insulin sensitivity or glucose tolerance. This supports the notion, also described by Chokshi and colleagues [29] that the occurrence of systemic insulin resistance in heart failure is linked to the regulation of cardiac Cer- and DAG-levels, a process not present in our model.

In addition, we found an increase of cardiac PIs in wt-TAC mice compared to sham mice, a regulation absent in atATGL-KO mice. PIs are important precursors of PI (4,5) biphosphate (PIP_2_), a crucial generator of two second messenger molecules, DAGs and inositol 1,4,5-triphosphate (IP_3_) [31]. Both signaling molecules have been shown to be involved in the pathogenesis of cardiac hypertrophy and heart failure [32, 33]. Thus, the upregulation of PIs in failing wt-hearts together with the absence of the regulation in atATGL-KO mice, may provide one potential additional underlying mechanism of the observed cardiac phenotype in our model.

The results described in this study are the first to demonstrate an induction of cardiac PE species during the development of LV failure. Furthermore, we showed that these cardiac lipidome changes were dependent on adipose tissue lipolysis. PEs are the most abundant phospholipids in mammalian cells, and are predominantly located in plasma membranes and mitochondrial membranes [34]. Disruption of whole-body PE synthesis in CTP:phosphoethanolamine cytidylyltransferase (Pcyt2) +/- mice resulted in myocardial hypertrophy and cardiac dysfunction in male mice [35]. However, these mice also developed a pronounced metabolic syndrome with abdominal obesity, hyperlipidemia, insulin resistance and liver steatosis which likely mediated the cardiac phenotype, thus making a direct comparison with our model difficult [35]. In this study, we found increased levels of PEs in human plasma samples and significantly higher levels of individual cardiac PE species during heart failure. Our data point towards a functional role of selected PE species in the pathogenesis of LV failure. Moreover, the reduction in circulating levels of distinct FAs in atATGL-KO mice predominantly affected the level of cardiac PE species, suggesting on the one hand that cardiac lipid species concentration is determined by plasma FA levels, and on the other hand that the FA distribution into different lipid classes occurs in a head group-specific manner. This process of cardiac FA partitioning into different cardiac lipid classes, and its functional relevance has recently gained greater attention [36]. In the context of pathological cardiac hypertrophy and failure, incorporation of oleate (C18:1) and linoleate (C18:2), but not other FAs, into cardiac DAGs has been associated with profound LV hypertrophy and systolic failure [36, 37]. In our analysis, DAG species were not significantly altered in failing wt-hearts, but two DAG species (16:0–18:2; 18:2–18:2) were elevated in plasma from HFrEF patients. Since DAGs are required for PE synthesis via the Kennedy pathway [34], the increase in cardiac tissue PE species observed in our study may be a potential consequence of higher circulating DAG levels. The fact that cardiac DAGs were not elevated in our model may suggest that our analysis was performed at a different time point during the pathogenesis of LV hypertrophy or failure compared to previous studies [36].

How does the increase in PE species affect the development of LV failure during pressure overload, and can the absence of this regulation explain why atATGL-KO mice are protected from LV failure? PEs are major constituents of plasma membranes [34]. Previous work in primary rat cardiomyocytes demonstrated that an increase of membrane PE content induces sarcolemmal destabilization and destruction [38]. Moreover, lowering the PE levels in cardiomyocytes, as seen for distinct PE-species in atATGL-KO mice, attenuated cardiomyocyte damage induced by ischemic or metabolic stress by maintaining the physicochemical properties of the sarcolemma [38]. Similarly, the ratio of PCs to PEs has been identified as a determinant of membrane integrity [17]. Decreased PC/ PE ratios induced membrane permeability in hepatocytes resulting in cell damage [17]. We calculated species-specific PC/PE ratios and discovered significant decreases accompanied with an increased Bax and cleaved caspase 3 expression only in failing wt-hearts but not in atATGL-KO hearts. Since we analyzed whole heart tissue we can currently not exactly define the cellular/ subcellular localization of the observed PC/PE ratio changes. There are several potential scenarios connecting the decreased PC/PE-ratio with the increased pro-apoptotic level of Bax/ cleaved caspase 3. Bax is known to control the intrinsic pathway of apoptosis promoting mitochondrial outer membrane (MOM) permeabilization and the release of pro-apoptotic factors [39]. In addition, changes of MOM´s lipid composition including a dysregulation of PE content or a decrease of the PC/PE ratio can affect the biogenesis of MOM proteins as well as Bax function [39, 40]. Thus, these data suggest that high cardiac PE levels and subsequent low PC/ PE ratios may affect MOM integrity thereby promoting Bax-mediated apoptosis. An alternative way may involve the extrinsic arm of apoptosis by which extracellular stress signals via death receptors initiate intracellular pro-apoptotic signaling. Death receptor signaling involves receptor redistribution into membrane lipid rafts, and lipid raft assembly is, at least in part, controlled by membrane lipid composition [41, 42]. Thus, one may hypothesize that changes of plasma membrane PC/PE content possibly augment death receptor signaling thereby promoting cardiomyocyte apoptosis, a process absent in atATGL-KO mice. However, future experiments are required to delineate the exact link between the decreased PC/PE ratios, increased Bax/ cleaved caspase 3 expression, and the impact on myocardial apoptosis.

An additional mechanism of how enhanced ATGL-activity/ AT-lipolysis and increased cardiac PE-levels may impair cardiac function might be the modulation of β-adrenergic signaling. Cardiac β2-adrenergic stimulation has been recently shown to be protective in CHF [43]. In parallel, phospholipids have been identified as G-protein-coupled receptor modulators, and in particular, increased levels of membrane PEs stabilize the β2-receptor in an inactive state [44]. Thus in addition to pro-apoptotic effects, the cardiac PE-increase in our study may have affected cardiac function by inhibiting protective cardiac β2-signaling.

We also identified changes in cardiac sphingolipid and CL levels. In particular, CLs have been implicated in the regulation of cardiac energy metabolism and function [45, 46]. A decrease in CLs, which are major mitochondrial membrane phospholipids, results in mitochondrial dysfunction with impaired oxidative phosphorylation, and has been associated with LV dysfunction [47]. In our study, we observed a reduction in CL 76:12 and it is possible that CL depletion may also have contributed to pressure-induced LV failure in our model.

Finally, changes in distinct plasma lipid classes and individual lipid species were shown in a small group of HFrEF patients when compared to a non-HFrEF control group. These data allude to the presence of lipidomic plasma changes in human heart failure which have been previously described in a metabolomics study [48]. In our study, the changes in selected PE species in human plasma corresponded to the changes in cardiac PE species observed in mice with LV failure, and this observation may be the first to suggest that PE dysregulation also occurs in human HFrEF pathogenesis. However, correlations between plasma/ serum and cardiac lipid compositions need to be judged very cautiously. For instance, the phospholipid composition varies markedly between plasma/ serum and tissues [49]. PEs, highly abundant in cardiac tissue, represent only 4% of the phospholipid fraction in plasma [50]. Tonks and colleagues investigated the correlation between plasma and muscle glycerolipids, sphingolipids, and cholesterol esters [51]. Of the 40 detected sphingolipid species in plasma and muscle only 8 correlated significantly [51]. This is further supported by a recent work from Ji and colleagues investigating the concentration of Cer species in serum and cardiac tissue samples from control and heart failure patients [52]. From 7 Cer species significantly regulated in serum only 3 exhibited significant changes in myocardial samples [52]. Together these data indicate a more complex relationship between tissue and plasma/ serum lipid composition. It is very likely that during the process of FA partitioning secreted FAs from adipose TAGs are incorporated into distinct plasma/ serum lipid classes which differ from the acceptor lipid class in target organs such as the heart [36].

Taken together, the results of this study demonstrate for the first time that perturbation of adipose tissue lipolysis by deletion of AT-specific ATGL leads to an improvement in pressure-induced heart failure, and can prevent potentially deleterious cardiac lipidome changes such as PE induction. Furthermore, we showed that the development of LV failure is associated with a distinct lipidomic profile in human plasma and mouse hearts, suggesting a process of cardiac FA partitioning into different cardiac lipid classes. Further studies are required to improve our understanding of the underlying mechanisms and functional consequences of cardiac FA partitioning during heart failure, and to identify novel therapeutic targets for the treatment of this disease.

## Materials and methods

### Animals

Male adipose tissue specific adipose triglyceride lipase (ATGL) deficient mice (atATGL-KO) and control littermates (wt) were generated, as described previously [12]. Mice were randomized to sham or transverse aortic constriction (TAC) operated groups. The TAC procedure was performed as previously described [53]. The detailed TAC protocol is provided in the S1 Supporting Information. Eleven weeks after surgery, mice were euthanized under isoflurane anesthesia by cervical dislocation.

All animal procedures were performed according to the guidelines of the Charité Universitätsmedizin Berlin and were approved by the Landesamt für Gesundheit und Soziales (Berlin, Germany) for the use of laboratory animals and according to the current version of the German Law on protection of animals.

### Echocardiography

Echocardiographic analysis was performed 11 weeks after sham/TAC-intervention, using Vevo 770 high-resolution imaging system with a 30-MHz transducer (RMV-707B; VisualSonics, Toronto, Canada) as described previously [12].

### Intraperitoneal glucose and insulin tolerance tests

Animals were metabolically phenotyped after 10 weeks. Intraperitoneal glucose tolerance tests (ipGTT) and intraperitoneal insulin tolerance tests (ipITT) were performed using a dose of 1 g/kg body weight (BW) glucose and by injecting 0.25 U/kg BW insulin (ActrapidHM, Novo Nordisk), respectively, as described previously [54]. Tail vein blood was used for glucose quantification during ipGTT and ipITT using a Glucometer (Precision Xtra, Abbott).

### HL-1 cell culture

Mouse HL-1 cardiomyocytes, kindly provided by W.C. Claycomb (Louisiana State University, LA) were cultivated and stimulated, as described previously [12]. Briefly, cells were starved for 24h and afterwards stimulated for 6 h with a mix of C16:0, C18:1 and C18:2, dissolved in 10% FFAs-free BSA. For experiments FAs were used in both equimolar- or 5x lower- serum concentration (FAmix and FAmix –low, respectively).

### mRNA analysis and western blotting

mRNA analysis and western immunoblotting were performed as previously described [55]. The details of these procedures are outlined in the S1 Supporting Information.

### Histology

Cardiac tissue samples were formalin-fixed, paraffin-embedded and stained with Hematoxylin/Eosin, and Picrosirius red, and analyzed, as previously described [12, 56].

Cardiac myocyte cross sectional area was assessed using H/E-stained sections of myocardium. Perimeter of the cell borders of 50 myocytes cut transversally were measured using cellSens (Olympus). Myocytes were selected in up to ten 400x microscopic fields each separated into 20 equally sized squares. Only one transversally myocyte per square was measured to increase representativeness. Fibrotic content (collagen fibers stained with picrosirius red) was assessed from the total cross section by a blinded expert in veterinary pathology using an analysis software (Olympus) and a semi-quantitative scoring system (0 = no fibrosis, 1 = occasionally collagen fibers (mild), 2 = several collagen fibers (moderate), 3 = massive accumulation of collagen fibers (severe)).

### Human blood samples

Human fasting plasma samples were collected from healthy subjects and patients with systolic heart failure (HFrEF) at the Division of Cardiology, University Hospital RWTH Aachen, Germany. Human plasma was isolated from EDTA blood samples by centrifugation at 2000rpm for 10min. Supernatant was collected and stored at -80°C as 200μL aliquots. Left ventricular (LV) ejection fraction was assessed by standard echocardiography. Blood sampling and clinical data collection and analysis was approved by an institutional review board (Ethics committee University Hospital RWTH, Aachen, Germany), and all participants provided written informed consent.

### FA- and TAG analysis of mouse blood and white adipose tissue samples

Mouse serum was isolated from whole blood. Blood samples were placed for 60min at room temperature for induction of coagulation. Afterwards, samples were centrifuged at 5000rpm for 5min at 4°C, and supernatant (serum) was collected. For FA serum profiling, 40μl samples of murine serum were hydrolyzed under alkaline-methanolic conditions, neutralized and diluted 1:10 in methanol containing internal FA standards. The HPLC measurement (Agilent 1200 HPLC system), coupled with an Agilent 6460 triple quad MS was performed, as described previously [12]. Serum concentration of FFAs was measured using a HR-NEFA kit (WAKO), as described previously [12]. TAGs in serum were determined using the DiaSys Triglycerides FS kit (DiaSys GmbH), according to the manufacturer’s instructions.

Lipids from adipose tissue were extracted using a two-step chloroform/ methanol procedure [57]. Samples were analyzed by direct infusion on a QExactive mass spectrometer (Thermo Scientific) equipped with a TriVersa NanoMate ion source (Advion Biosciences). Further details see section Lipidomic Analysis.

### Lipidomic analysis

Lipid extraction and analysis of mouse LV samples (3mg per sample) and human plasma (15μl per sample) were performed as described previously [58, 59] at Lipotype GmbH: Lipids were extracted using a two-step chloroform/methanol procedure [57]. Samples were spiked with internal lipid standard mixture containing: cardiolipin 16:1/15:0/15:0/15:0 (CL), ceramide 18:1;2/17:0 (Cer), diacylglycerol 17:0/17:0 (DAG), hexosylceramide 18:1;2/12:0 (HexCer), lyso-phosphatidate 17:0 (LPA), lyso-phosphatidylcholine 12:0 (LPC), lyso-phosphatidylethanolamine 17:1 (LPE), lyso-phosphatidylglycerol 17:1 (LPG), lyso-phosphatidylinositol 17:1 (LPI), lyso-phosphatidylserine 17:1 (LPS), phosphatidate 17:0/17:0 (PA), phosphatidylcholine 17:0/17:0 (PC), phosphatidylethanolamine 17:0/17:0 (PE), phosphatidylglycerol 17:0/17:0 (PG), phosphatidylinositol 16:0/16:0 (PI), phosphatidylserine 17:0/17:0 (PS), cholesterol ester 20:0 (CE), sphingomyelin 18:1;2/12:0;0 (SM), triacylglycerol 17:0/17:0/17:0 (TAG). After extraction, the organic phase was transferred to an infusion plate and dried in a speed vacuum concentrator. 1st step dry extract was re-suspended in 7.5 mM ammonium acetate in chloroform/methanol/propanol (1:2:4, V:V:V) and 2nd step dry extract in 33% ethanol solution of methylamine in chloroform/methanol (0.003:5:1; V:V:V). All liquid handling steps were performed using Hamilton Robotics STARlet robotic platform with the Anti Droplet Control feature for organic solvents pipetting.

Samples were analyzed by direct infusion on a QExactive mass spectrometer (Thermo Scientific) equipped with a TriVersa NanoMate ion source (Advion Biosciences). Samples were analyzed in both positive and negative ion modes with a resolution of Rm/z = 200 = 280000 for MS and Rm/z = 200 = 17500 for MSMS experiments, in a single acquisition. MSMS was triggered by an inclusion list encompassing corresponding MS mass ranges scanned in 1 Da increments [59]. Both MS and MSMS data were combined to monitor CE, DAG and TAG ions as ammonium adducts; PC, PC O-, as acetate adducts; and CL, PA, PE, PE O-, PG, PI and PS as deprotonated anions. MS only was used to monitor LPA, LPE, LPE O-, LPI and LPS as deprotonated anions; Cer, HexCer, SM, LPC and LPC O- as acetate adducts. Data were analyzed with in-house developed lipid identification software based on LipidXplorer [60, 61]. Only lipid identifications with a signal-to-noise ratio >5, and a signal intensity 5-fold higher than in corresponding blank samples were considered for further data analysis.

### Bioinformatic data analysis and statistics

Measured lipid amounts were normalized to total lipid amount in samples and log-transformed to approach a symmetric and approximative normal distribution. Lipid species with missing values across many samples were removed from further analysis, and in total, we evaluated the results of 225 lipid species in the mouse and 147 in the human data set. Normal distribution was checked visually by using Q-Q plots. Depending on their scale and distribution, results are given in proportions, mean, standard deviation or standard error of the median with 25% to 75% quartiles as indicated. Statistical tests for significance were performed by using the tow-tailed t-test or Mann-Whitney-U-test as appropriate. Multivariable analysis was done by linear and robust linear regression with log-transformed relative lipid amounts as dependent and experimental or clinical conditions, age and BMI, as independent variables. For class level comparisons only, we used regression or median imputation of missing values. To avoid alpha inflation, p-values were adjusted by the Benjamini-Hochberg procedure [62]. Adjusted p-values of less than <0.1 were considered to be significant. All analyses were performed with R version 3.3.1. See S1 Supporting Information for further details regarding data analysis.

To compare differences between groups of mice phenotypes, we performed two-way ANOVA (Bonferroni posttest), two-way ANOVA with repeated measures (Bonferroni posttest) or t-test, as appropriate, and data was analyzed with GraphPad Prism Software. Statistical significance was assumed at p<0.05. Vertical lines in the histograms indicate means ± standard error of the mean (SEM). The n-number is indicated for each experiment.

## Supporting information

S1 Text (Material and methods): A detailed description of the used methods and materials is shown. (DOCX)

S1 Table Cardiac phenotype/ echocardiographic analysis of atATGL-KO mice and wt-littermates, 11 weeks after TAC/ sham surgery. (DOCX)

S2 Table MS-based shotgun lipidomics analysis of heart tissue samples (LV) isolated 11 weeks after intervention (sham or TAC) from wild-type (wt) or atATGL-KO mice. Shown are raw data lipid concentrations in pmole.
(CSV)

S3 Table PC/ PE-ratios calculated from raw data presented in S2 Table. (CSV)

S4 Table ID, age (years) and body mass index (BMI) from HFrEF (n = 13) and non-HFrEF (n = 10) patients. (CSV)

S5 Table MS-based shotgun lipidomics analysis of human plasma samples from HFrEF-patients (n = 13) and non-HFrEF controls (n = 10). Shown are raw data lipid concentrations in pmole.
(CSV)

S1 Fig **A**: ATGL mRNA expression in cardiac endothelial cells. Cardiac endothelial cells were isolated from cardiac tissue of atATGL-KO- and wt-mice using a Langendorff perfusion system, collagenase Type II digestion and CD31 microBeads Macs Miltenyi Biotec System, as described in methods. qRT-PCR studies of ATGL expression, relative to 18S were carried out using total RNA isolated from those cells (mean and SEM, n = 3–4, n.s.: statistical non-significant). **B:** MS-based shotgun lipidomics analysis of Triacylglycerol (TAG) species in white adipose tissue samples (WAT) isolated 11 weeks after intervention (sham) from wt- and atATGL-KO mice. *p<0.05 vs. wt sham. **C:** Profile of selected serum FAs in sham-operated mice analyzed by rapid resolution HPLC/ Tandem MS. (mean and SEM, n = 5, unpaired ttest). *p<0.05 vs. wt sham. **D:** WB analysis of HL-1 cells cultivated in starving (0.5% FBS) or full medium (10% FBS), using antibodies against cleaved caspase 3, loading control: beta-Actin (βActin).
(TIF)
