# Supplementary material for: Adipose tissue ATGL modifies the cardiac lipidome in pressure-overload-induced left ventricular failure
Source: PLoS Genet. 2018 Jan 10;14(1):e1007171. doi: 10.1371/journal.pgen.1007171 (PMC5779697; doi:10.1371/journal.pgen.1007171)
Supplement: S1 Text — (DOCX) [file pgen.1007171.s001.docx]

**SUPPORTING INFORMATION**

**FULL TITLE:**

**Adipose Tissue ATGL Modifies the Cardiac Lipidome in Pressure-Overload-Induced Left Ventricular Failure**

Supplemental Material and Methods

**Supplemental Material and Methods**

**Animals**

The mice were housed under constant environmental conditions: temperature 20–24°C, 55 ± 10% relative humidity, 12-h:12-h light-dark cycle, and fed at libitum with a standard diet. Eight-9 week old atATGL-KO- and wt-mice were then randomized to sham or TAC (transverse aortic constriction) operated groups. Body weight (BW) development was monitored throughout the experiment. Eleven weeks after surgery, mice were euthanized under isoflurane anesthesia by cervical dislocation. Final blood samples were collected and organs were dissected and snap frozen in liquid nitrogen and stored at −80°C. For histological analysis tissue, samples were placed in 10% formalin solution for up to 24 h, washed in PBS-buffer, and embedded in paraffin.

**Transverse Aortic Constriction**

Mice were anesthetized by intraperitoneal injection of ketamine (Ketanest, 100 mg/kg), xylazine/HCl (Rompun, 20 mg/kg) and acepromazine (3mg/kg) solution. Next, mice were placed in the supine position under a surgical microscope, intubated via an endotracheal approach and connected to a rodent ventilator (MiniVent Model 845 Ventilator, Hugo Sachs/March-Hugstetten). Sternotomy was subsequently performed, and the aorta and carotid arteries were exposed. The transverse aorta was then ligated by tying a 6-0 silk suture (FST) against a 26-gauge needle. The needle was then removed to yield the constriction with a diameter of approximately 0.4 mm. Finally, the chest was closed with a 7-0 silk suture. Sham animals underwent an identical surgical procedure without placement of the suture. All mice were given post-operative Rimadyl (5 mg/kg, s.c.) analgesia, and kept under a red-light lamp and normal ventilation. After recovery from the operation, the animals were intensively monitored. Throughout the first post-operative week, mice were given daily Rimadyl (5 mg/kg, s.c.) or metamizole solution (200mg/kg) dissolved in drinking water.

Success of the ligation at the transverse aorta was assessed by measuring pressure gradients proximal and distal of the ligation. Pressure gradients were calculated from blood flow velocities assessed by Doppler Echocardiography using the Bernoulli equation [1] .

**Echocardiography**

Echocardiographic analysis was performed 11 weeks after sham/TAC-intervention, using Vevo 770 high-resolution imaging system with a 30-MHz transducer (RMV-707B; VisualSonics, Toronto, Canada). The mice were initially anesthetized with 3% isoflurane (inhalation), fixed on a heated pad at 37°C and continuously monitored by ECG recording. During the analysis, isoflurane was reduced to 1.5-2%. Scans were performed in the parasternal short axis of the heart in B- and M-mode. The thickness of the posterior and anterior wall, as well as the intraventricular diameter, was determined in both systole and diastole. We calculated left ventricular mass (LVM),ejection fraction (EF%), and fraction shortening (FS%).

**Isolation of cardiac endothelial cells**

Ventricular cardiac cells were isolated as previously described [2]. Briefly: mice were injected with heparin (10.000 I.E/kg i.p.). Afterwards the animals were anesthetized with isoflurane (Abbott, Wiesbaden, Germany), and killed by cervical dislocation. The heart was removed and perfused on a Langendorff apparatus using Ca^2+^-free Tyrode's solution with taurine (15 mmol/L). Cardiac tissue was digested with collagenase Type II (Worthington, Lakewood, NJ, USA) and mechanical dispersed. Next, cardiac cells were centrifuged (500rpm/1min/RT), and the cell pellet containing cardiomyocytes was removed. Remaining supernatant was subjected to the isolation of cardiac endothelial cells, using the Macs Miltenyi Biotec system, consisting of CD31 MicroBeads and LS Columns, according to manufacturer’s suggestions. Briefly: cardiac cells were centrifuged (300rpm, 10 min, +4°C), washed with washing buffer (containing PBS, 0.5% FA-free BSA and 2mM EDTA) and incubated with CD31 MicroBeads for 15 min on ice. Afterwards the cells were washed, centrifuged, re-suspended in washing buffer, and separated on the LS Column, placed in the magnetic field. After several washing steps, CD31 positive cells were eluted and pelleted by centrifugation. Pelleted cells were used for a total RNA isolation, performed with microRNA-KIT (Qiagen).

**mRNA Analysis**

Total RNA from heart was isolated using Qiazol and the RNAeasy Micro Kit from Qiagen according to the manufacturers' instructions. LV tissue samples were lysed using Lysis Tubes P and Speed-Mill system (Anatik Jena; Biometra) following digestion with proteinase K. The RNA was DNAse digested with RNAse free DNAse set (Qiagen) and reverse transcribed using reverse transcriptase, RNAsin, and dNTPs (Promega) according to the manufacturers' instructions. mRNA analysis was performed using quantitative RT-PCR analysis in the presence of SYBR-Green fluorescent dye (Life Sciences). The qRTPCR results were normalized to 18S and beta-Actin. The primer sequences are available on demand.

**Western Blotting**

For western blot (WB) analysis, the LV tissue samples were lysed in RIPA buffer (50 mM Tris pH 7.5, 150 mM NaCl, 5 mM MgCl_2_, 1% Nonidet P-40, 2.5% glycerol, 1 mM EGTA, 50 mM NaF, 1 mM Na_3_VO_4_, 10 mM Na_4_P_2_O_7_, 100 µM phenylmethylsulfonyl fluoride and complete protease/ phosphatase inhibitor cocktail (Phos-stop and Complete Mini, Roche Diagnostics) using Lysis Tubes P (Anatik Jena; Biometra) and Speed-Mill system, analogous to the RNA-extraction protocol. Lysates were analyzed by immunoblotting using antibodies raised against Bcl-associated X protein (Bax, #14796, Cell Signaling Technologies), cleaved caspase 3 (#9664, Cell Signaling Technologies), Glyceraldehyde 3-phosphate dehydrogenase (GAPDH, Abcam), β-actin (Santa Cruz, SC81178) and secondary horseradish-conjugated antibodies (Jackson Immuno Research). For detection, enhanced chemiluminescent reagents (ECL kit, Thermo Scientific) were used.

**Bioinformatic data analysis**

**Processing of mouse heart data:**

One of the 22 mouse heart data sets was removed due to its large number of missing values. The remaining data sets were normalized with respect to the total amount of measured lipids and values were log2 transformed, yielding ${log}_{2}\left( r \right)$, where $r$ is the relative change. Species with less then three valid measurements in any condition were excluded from the further analysis. This process resulted in a final count of 225 lipid species from an initial 542.

To filter for differentially regulated lipid species we used a linear model,

$${log}_{2}\left( r \right)=\mu+trt+ko+trt:ko.$$

$\mu$ denotes the offset of the measured value as given by wt_sham, and $trt:ko$ denotes the interaction between treatment, $trt$, and $ko$. Next we tested for the hypotheses that

- $trt=0$, i.e. there is no effect due to the treatment
- $ko=0$, i.e. there is no effect due to the genotype
- $trt+ko+trt:ko=0$, i.e. the interaction restores the wt level without treatment

For each test we obtained the t-statistic, and multiple testing corrections were applied to obtain adjusted p-values using the function glht from the multcomp R package. Since we did this for each lipid species, we also applied multiple testing corrections when filtering for differentially regulated species. To that end, we determined FDR adjusted p-values and retained all species which had an $adjusted p-value<0.1$ in at least one hypothesis test. We used the t-statistics to cluster the filtered lipid species with hierarchical clustering using the complete linkage method with a Euclidean distance. Four different clusters were identified by cutting the tree at a height of 8. The resulting clustering was used to order the mean log2 transformed normalized data, ${log}_{2}\left( r \right)$, as shown in the corresponding heat map.

Using t-tests, we also tested directly for differentially regulated species between wt sham and wt TAC, and ko sham and ko TAC, respectively. Again, FDR adjusted p-values were calculated. We classified a species as significantly changed if $adjusted p-value<0.1$ and $|\overline{{log}_{2}\left( r_{tac} \right)}-\overline{{log}_{2}\left( r_{sham} \right)}|=|{log}_{2}fc|\geq0.5.$ Here the over-line denotes the mean across all samples from a condition. In addition, we calculated the mean mole percent by averaging the mole percent estimates over all samples from both conditions.

In order to test whether specific PC/PE ratios are changed, we grouped PC and PE species based on their fatty acids. We then performed a Mann-Whitney U test for each pair and compared wt-sham ratios against wt-tac, and ko-sham against ko-tac. After correcting for multiple testing we retained those ratios with a FDR $adjustedp-value<0.1$.

Next we compared the lipid changes on the class level. Here we applied an imputation strategy to deal with the missing values. To that end, we grouped species by condition and substituted missing values by the observed median. For each sample we then summed up the mole percent values, $2^{{log}_{2}\left( r \right)}\times100$, of all species in a class. Finally, we calculated the mean and standard error of mean in each condition, and used a Mann-Whitney U test to filter for differentially regulated classes upon intervention in each genotype. Multiple testing corrections were applied by calculating FDR adjusted p-values.

**Processing of data from human blood analysis**

The clinical data comprised 23 samples obtained from HFrEF-patients (n=13) and healthy controls (n=10). Again, we normalized by the total amount of measured lipid and log2 transformed the normalized data. Species with less than 6 valid measurements in the control or the patient group were excluded from the further analysis. This resulted in a total of 147 lipid species from an initial 209 lipid species. In order to control for age and body mass index (BMI), we used the following linear model:

$${log}_{2}\left( r \right)=\mu+group+age+bmi,$$

where $\mu$ denotes the offset for the healthy control group. As we are interested in the disease effect, we corrected the p-values for the group coefficient estimates using a FDR adjustment. In order to account for potential outliers we used the robust linear regression, lmRob from the robust R package. Species with an $adjusted p-value<0.1$ and an offset of at least 0.5 between groups were classified as differentially regulated. Errors are standard errors from the linear regression. In order to determine the scale at which the observed fold change takes place, we used the linear model regression coefficients to estimate the mole percent of each species in the healthy control at the mean study age (~ 52) and BMI (~ 26.5).

In order to estimate the usage of each class, we imputed missing values. To do this, we used the regression parameters to estimate missing values depending on the group, age, and BMI. In a second step, we corrected for age and BMI by calculating

$${log}_{2}\left( \rho\right)={log}_{2}\left( r \right)-\Delta age-\Delta bmi,$$

such that all values are with respect to the mean age and BMI. Consequently we removed the one individual for which no BMI data was available. We then summed up the mole percent values, $2^{{log}_{2}\left( \rho\right)}\times100$, of all species in a class.


**References**

1. Richards KL. Assessment of aortic and pulmonic stenosis by echocardiography. Circulation. 1991;84(3 Suppl):I182-7. PubMed PMID: 1884484.

2. Antoons G, Mubagwa K, Nevelsteen I, Sipido KR. Mechanisms underlying the frequency dependence of contraction and [Ca(2+)](i) transients in mouse ventricular myocytes. J Physiol. 2002;543(Pt 3):889-98. PubMed PMID: 12231646.
