# Supplementary material for: Adipose tissue ATGL modifies the cardiac lipidome in pressure-overload-induced left ventricular failure
Source: PLoS Genet. 2018 Jan 10;14(1):e1007171. doi: 10.1371/journal.pgen.1007171 (PMC5779697; doi:10.1371/journal.pgen.1007171)
Supplement: S1 Table — (DOCX) [file pgen.1007171.s002.docx]

**S1 Table. Cardiac phenotype of atATGL-KO mice and wt littermates 11 weeks after TAC/sham surgery.**

|  | WT Sham | WT TAC | atATGL-KO Sham | atATGL-KO TAC |
| --- | --- | --- | --- | --- |
| Echocardiography: |  |  |  |  |
| LVM | 90.95 ± 5.4 | 211.16 ± 19.73**** | 94.60 ± 2.92 | 124.32 ± 9.06^$$$$^ |
| LVM/TL | 7.44 ± 0.47 | 17.06 ± 1.50**** | 7.88 ± 0.23 | 10.53 ± 0.70^$$$^ |
| IVS-d [mm] | 0.59 ± 0.01 | 0.90 ± 0.01**** | 0.60 ± 0.01^####^ | 0.76 ± 0.03^$$$^ |
| LVPW-d [mm] | 0.60 ± 0.01 | 0.87 ± 0.01**** | 0.59 ± 0.01 | 0.75 ± 0.03^$$$^ |
| LVID-d [mm] | 4.32 ± 0.11 | 5.28 ± 0.24*** | 4.40 ± 0.06 | 4.31 ± 0.11^$$$$^ |
|  |  |  |  |  |
| Other parameters: |  |  |  |  |
| HW/ TL [mg/mm] | 10.56 ± 0.29 | 18.08 ± 0.97**** | 10.09 ± 0.5^#^ | 13.01 ± 0.66^$$$$^ |
| HR [beats/min] | 440.3 ±14.41 | 446.0 ± 13.07 | 407.4 ± 14.58 | 470.4 ± 27.82 |

Left-ventricular mass (LVM); Left-ventricular mass relative to tibia length (LVM/TL) Interventricular septum thickness in diastole (IVS-d); Left-ventricular posterior wall thickness in diastole (LVPW-d); Left-ventricular internal diameter in diastole (LVID-d); Heart weight (gravimetric) to tibia length (HW/TL); Heart rate (HR); Mean and SEM, n= 7, ***p<0.001 vs. wt sham, ****p<0.0001 vs. wt sham,^$$$^ p<0.001 vs. wt TAC, ^$$$$^ p<0.0001 vs. wt TAC, ^#^p<0.05 vs. atATGL-KO TAC, ^####^p<0.0001 vs. atATGL-KO TAC; 2-way ANOVA (Bonferroni posttest).
