# Supplementary figures and images for: Adipose tissue ATGL modifies the cardiac lipidome in pressure-overload-induced left ventricular failure
Source: PLoS Genet. 2018 Jan 10;14(1):e1007171. doi: 10.1371/journal.pgen.1007171 (PMC5779697; doi:10.1371/journal.pgen.1007171)

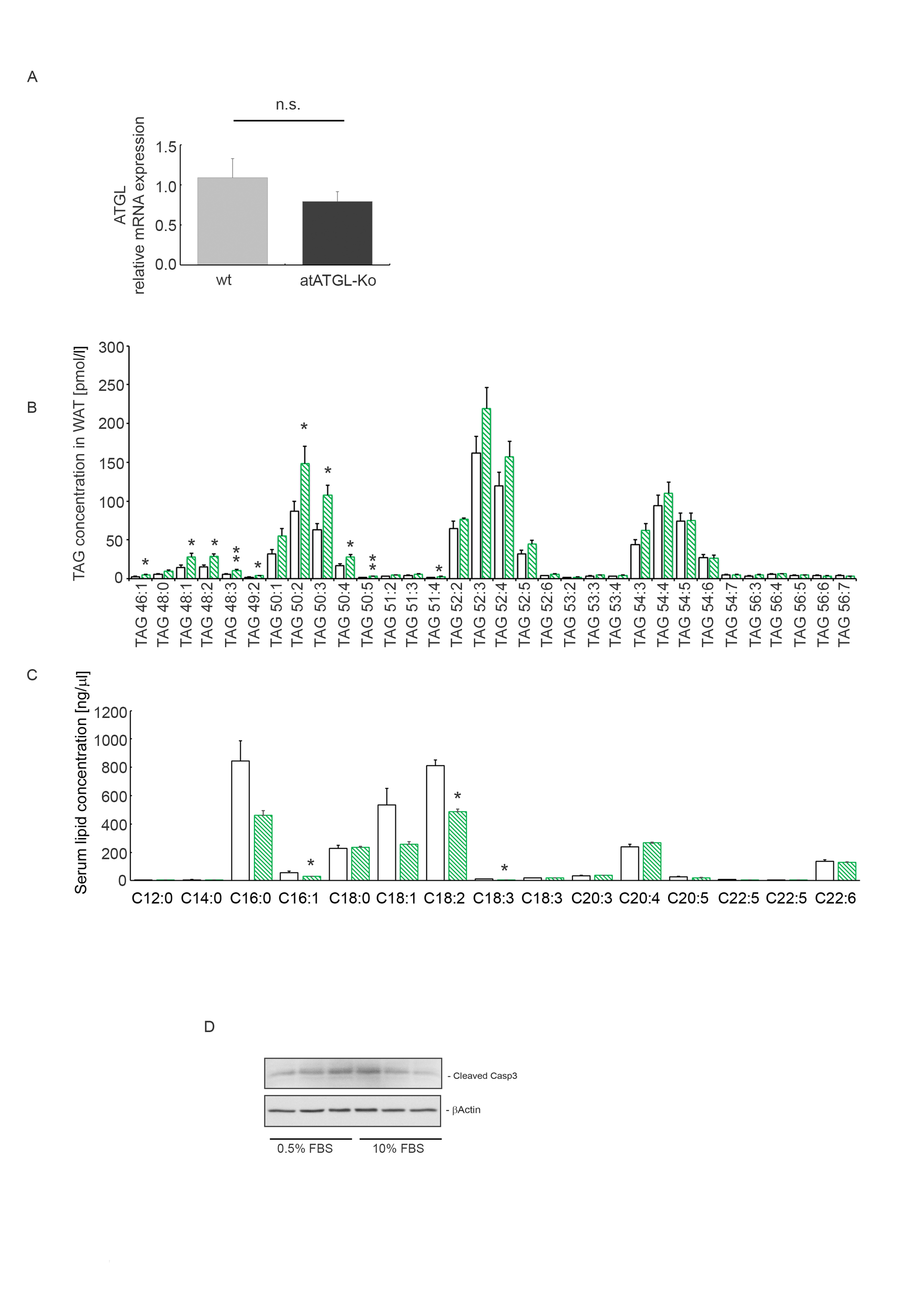

Supplement: S1 Fig — A: ATGL mRNA expression in cardiac endothelial cells. Cardiac endothelial cells were isolated from cardiac tissue of atATGL-KO- and wt-mice using a Langendorff perfusion system, collagenase Type II digestion and CD31 microBeads Macs Miltenyi Biotec System, as described in methods. qRT-PCR studies of ATGL expression, relative to 18S were carried out using total RNA isolated from those cells (mean and SEM, n = 3–4, n.s.: statistical non-significant). B: MS-based shotgun lipidomics analysis of Triacylglycerol (TAG) species in white adipose tissue samples (WAT) isolated 11 weeks after intervention (sham) from wt- and atATGL-KO mice. *p<0.05 vs. wt sham. C: Profile of selected serum FAs in sham-operated mice analyzed by rapid resolution HPLC/ Tandem MS. (mean and SEM, n = 5, unpaired ttest). *p<0.05 vs. wt sham. D: WB analysis of HL-1 cells cultivated in starving (0.5% FBS) or full medium (10% FBS), using antibodies against cleaved caspase 3, loading control: beta-Actin (βActin). (TIF) [file pgen.1007171.s007.tif]
